# Identification of a common reference gene pair for qPCR in human mesenchymal stromal cells from different tissue sources treated with VEGF

**DOI:** 10.1186/1471-2199-15-11

**Published:** 2014-05-28

**Authors:** Josefine Tratwal, Bjarke Follin, Annette Ekblond, Jens Kastrup, Mandana Haack-Sørensen

**Affiliations:** 1 Cardiology Stem Cell Centre, The Heart Centre, Rigshospitalet, Copenhagen University Hospital, Juliane Maries Vej 20, dept. 9302, 2100 Copenhagen, Denmark

**Keywords:** Adipose-derived stromal cell, ASC, ADSC, MIQE, qPCR, Reference gene, Mesenchymal stromal cell, MSC, Vascular endothelial growth factor, VEGF

## Abstract

**Background:**

Human mesenchymal stromal cells from the bone marrow (BMSCs) are widely used as experimental regenerative treatment of ischemic heart disease, and the first clinical trials using adipose-derived stromal cells (ASCs) are currently being conducted. Regenerative mechanisms of BMSCs and ASCs are manifold and *in vitro* pretreatment of the cells with growth factors has been applied to potentially enhance these properties. When characterizing the transcriptional activity of these cellular mechanisms *in vitro* it is important to consider the effect of the growth factor treatment on reference genes (RGs) for the normalization of qPCR data.

**Results:**

BMSCs and ASCs were stimulated with vascular endothelial growth factor A-165 (VEGF) for one week, and compared with un-stimulated cells from the same donor. The stability of nine RGs through VEGF treatment as well as the donor variation was assessed using the GenEx software with the subprograms geNorm and Normfinder.

The procedure of stepwise elimination was validated by poor performance of eliminated RGs in a normalization experiment using *vWF* as target gene. Normfinder found the TATA box binding protein *(TBP)* to be the most stable single RG for both BMSCs and ASCs. The optimal number of RGs for ASCs was two, and the lowest variance for *vWF* normalization was found using *TBP* and *YWHAZ*. For BMSCs, the optimal number of RGs was four, while the two-RG combination producing the most similar results was *TBP* and *YWHAZ*.

**Conclusions:**

A common reference gene, *TBP*, was found to be the most stable standalone gene, while *TBP* and *YWHAZ* were found to be the best two-RG combination for qPCR analyses for both BMSCs and ASCs through the VEGF stimulation. The presented stepwise elimination procedure was validated, while we found the final normalization experiment to be essential.

## Background

Human bone marrow derived stromal cells (BMSCs) and adipose-derived stromal cells (ASCs) have made a significant impact on the field of regenerative medicine. Within the field of cardiac regeneration, BMSCs have been used frequently in clinical trials while the first clinical trials with ASCs stimulated with vascular endothelial growth factor (VEGF) is currently being conducted by our group [1,2]. BMSCs are the most extensively investigated cell type, however adipose tissue has received increasing interest as a source of stem cells due to easier harvest, a larger yield of stem cells and the fact that ASCs possess higher proliferative capacity compared to BMSCs [3]. The rationale behind the potential benefit of using the two cell types has primarily been suggested on the basis of their immunoregulatory properties, paracrine profiles, and capability to differentiate into various cell types [4-9]. When investigating these properties and how to enhance them *in vitro*, the first change within the cells is found at the transcriptional level. This change is detectable by quantitative real time polymerase chain reaction (qPCR) analyses, which is the preferred method for detecting changes in gene expression [10]. However, the qPCR method is sensitive to several errors in the experimental setup that may affect results. These errors could occur at different stages throughout the experiment and encompass variations in the amount of starting material, nuclear extraction, RNA integrity, cDNA loading, reverse transcription, and qPCR efficiency [11]. Thus, in order to compare qPCR results from different runs, or samples obtained from different tissues at various time points, a normalization step is necessary [12].

The most widely used method for normalizing qPCR data is comparison to values from reference genes (RGs) analyzed simultaneously on the same material [13]. The abundance of the normalization candidate gene must be closely related to the total amount of mRNA in the sample, in order to be able to correct for experimental errors [14]. In addition, it must exhibit stable expression during experimental conditions to be useful for normalization. Often an invariant endogenous gene is used as a RG with a constant expression level across all samples and experimental treatments. If the RGs are not stable through the treatment it could obscure actual changes and produce artifacts that contribute to misleading results and incorrect conclusions, thereby leading to unreliable publications [13]. The attention regarding selection of suitable RGs has increased in the past decade, from the geometric mean normalization proposed by *Vandesompele et al.*, to experiment-specific RG investigations, and ultimately the “Minimum Information for Publication of Quantitative Real-Time PCR Experiments” (MIQE) guidelines [14,15]. The guidelines function as a universal checklist that investigators can follow, to produce the most reliable data when using qPCR [10].

In the field of cardiac regeneration, it has been proposed that endothelium-committed cells greatly enhance cell therapy outcome [16]. Ongoing clinical trials performed by our group serve to differentiate ASCs and BMSCs toward endothelial lineage for enhancement of angiogenic potential by stimulation with vascular endothelial growth factor (VEGF) [2,17,18]. This pro-angiogenic growth factor is known to promote endothelial cell proliferation, migration, and vessel permeability, and to differentiate BMSCs toward endothelial lineage [19,20]. The impact of VEGF treatment on ASCs and BMSCs is tested *in vitro* by the use of qPCR, where it is very important for both cell types to have established RGs that are stable through the VEGF treatment. A suitable RG in ASCs is not necessarily stable in BMSCs, therefore, it is important to validate whether the chosen RGs can be used in both cell sources under specific experimental conditions. No studies comparing choice of RGs between ASCs and BMSCs have been conducted previously to the authors’ knowledge, which makes this study particularly interesting with regard to the ongoing debate of differences and similarities between these two cell types. Through this study we provide a simple stepwise selection procedure for choosing the RGs with focus on the impact on the final outcome. For the purpose we use specially designed software GenEx, more specifically the algorithms Normfinder and geNorm [14,21].

## Results

**Figure 1:**
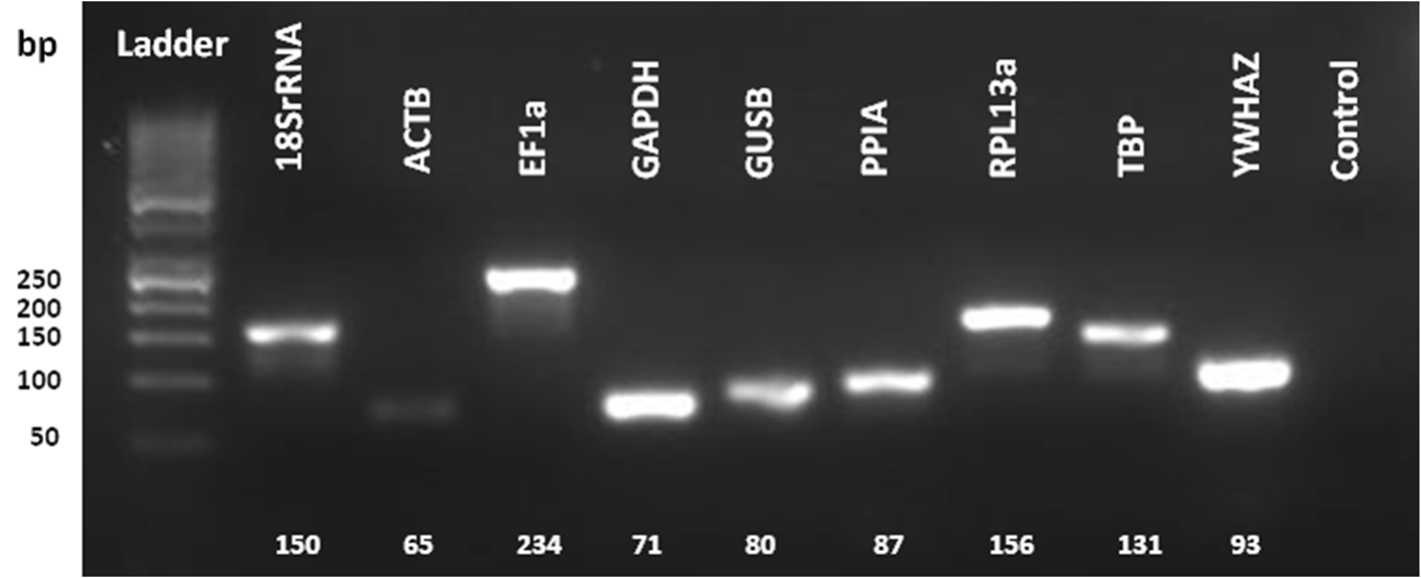
**Performance of primer amplification.** qPCR endpoint products were separated by agarose gel electrophoresis. Target genes are indicated above the amplicon.

To be able to apply the qPCR on VEGF pre-conditioned human mesenchymal stromal cells from the bone marrow and adipose tissue, a panel of RGs (Table 1) from relevant literature was chosen to find the correct RG for normalization. After confirming RNA purity, RNA integrity, primer efficiency, and products (Figure 1), a stepwise elimination of candidate RGs was performed in order to select the best-suited RG(s) from a list of acceptable ones.

**Table 1:** Reference genes for qPCR

**Gene**	**Gene**	**NCBI reference sequence**	**Forward sequence**	**Cellular function**
*18SrRNA*	18S ribosomal RNA	NR_003286.2	F = 5’-GTAACCCGTTGAACCCCATT-3’	Small subunit of cytoplasmic ribosomes
			R = 5’-CCATCCAATCGGTAGTAGCG-3’	
*ACTB*	beta-actin	NM_001101.3	F = 5’-CCTTTTTGTCCCCCAACTTGA-3’	Cytoskeleton structural protein; motility, cytokinesis
			R = 5’-TGGCTGCCTCCACCCA-3’	
*EF1a*	elongation factor-1 alpha	NM_001402.5	F = 5’-AGGTGATTATCCTGAACCATCC-3’	Translation
			R = 5’-AAAGGTGGATAGTCTGAGAAGC-3’	
*GAPDH*	glyceraldehyde 3-phosphate dehydrogenase	NM_002046.4	F = 5’-CAACGGATTTGGTCGTATTGG-3’	Oxido-reductase in glycolysis and gluconeogenesis, transcription activation, apoptosis
			R = 5’-GCAACAATATCCACTTTACCAGAGTTAA-3’	
*GUSB*	beta-glucuronidase	NM_000181.3	F = 5’-CTCATTTGGAATTTTGCCGATT-3’	Catalyzing hydrolysis of B-D-glucuronic acid
			R = 5’-CCGAGTGAAGATCCCCTTTTTA-3’	
*PPIA*	peptidyl prolyl isomerase A	NM_021130.3	F = 5’-TCCTGGCATCTTGTCCATG-3’	Protein folding
			R = 5’-CCATCCAACCACTCAGTCTTG-3’	
*RPL13*	ribosomal protein L13a	NM_012423.3	F = 5’-CATAGGAAGCTGGGAGCAAG-3’	Structural component of 60S ribosomal subunit
			R = 5’-GCCCTCCAATCAGTCTTCTG-3’	
*TBP*	TATA-binding protein	NM_003194.4	F = 5’-TGCACAGGAGCCAAGAGTGAA-3’	RNA polymerase II transcription factor
			R = 5’-CACATCACAGCTCCCCACCA-3’	
*YWHAZ*	tyrosine 3-monooxygenase/tryptophan 5-monooxygenase activation protein, zeta polypeptide	NM_001135702.1	F = 5’-ACTTTTGGTACATTGTGGCTTCAA-3’	Signal transduction
			R = 5’-CCGCCAGGACAAACCAGTAT-3’	

Information for the panel of selected reference genes with full name, NCBI reference sequence, forward and reverse primer sequences, and short description of cellular function.

### RNA and primer quality

RNA purity was validated by excluding protein contamination with absorbance ratios of 1.8-2.2 at 260 nm/280 nm. RNA integrity was confirmed by RIN values of 10 for all donors (Additional file 1: Table S1). Primers (Table 1) for the qPCR were validated by dissociation curves (Additional file 1: Figure S1) and by efficiencies ($E=\left(10^{\frac{-1}{\mathrm{slope}}}-1\right)∙100)$ of 100% ±10% with a correlation coefficient R^2^ between 0.985 and 1.0 (Additional file 1: Table S2).

### RG candidate selection

#### Step 1: RG candidate expression levels

**Figure 2:**
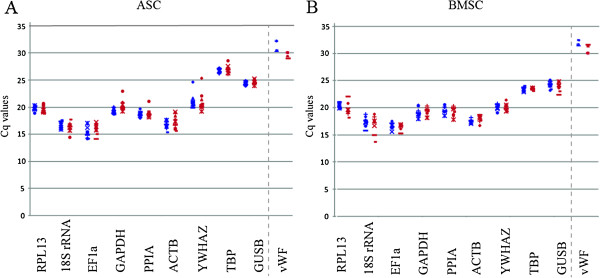
**Expression level dot plots of RG candidates.** Distribution of median Cq values for ASCs **(A)** and BMSCs **(B)** from eight donors in control medium (blue) and VEGF-treatment medium (red). ASCs; adipose-derived stromal cells, BMSCs; bone marrow-derived stromal cells, RG; reference gene, VEGF; vascular endothelial growth factor. N = 8 except for *vWF* where N = 3.

The RG candidate Cq values were generally between 15 and 25 cycles, with no great difference between the VEGF treatment group and the control group for either ASCs or BMSCs (Figure 2). The selected RGs should preferably be expressed in amounts close to those of the target genes. The raw data for the outliers provided us with Cq values that were substantially lower than that of the gene of interest, as depicted in Figure 2 presenting median Cq values. Reference genes with a substantially greater difference in abundance compared to the gene of interest are not suitable. It would be incorrect to perform an analysis on putative reference genes that are unsuitable to be considered. Here our cutoff Cq value difference is 10 cycles. In order not to reduce the initial panel of reference genes completely, we didn’t exclude more than the three genes mentioned at this stage.

We have investigated the impact of VEGF treatment in terms of initiation of endothelial differentiation in ASCs and BMSCs, after which endothelial target genes such as *vWF* are expressed in relatively low amounts, and therefore detected around 30 cycles. Since *EF1-α*, *ACTB*, and *18S rRNA* were the most abundant RGs in both ASCs and BMSCs, with Cq values far from our target, they were removed from the panel (Figure 2). This initial exclusion resulted in the six RGs expressed in closest abundance to the target gene remaining for further analyses.

#### Step 2: Initial standard deviation-values from Normfinder

Normfinder selects the most suitable RG based on the standard deviations (SDs) for intragroup donor variation and intergroup treatment variation. This analysis was performed taking the group classification of treatment into account, in order to verify that the candidate RGs were stably expressed and not regulated by treatment. The RG candidates with a large bias, an intergroup SD reaching or exceeding 0.2 were excluded (Table 2), leaving *TBP*, *PPIA*, *YWHAZ*, and *GUSB* for ASCs and *TBP*, *PPIA*, *YWHAZ*, *GUSB*, and *GAPDH* for BMSCs for the final analysis.

**Table 2:** Step two of elimination, showing ASC and BMSC reference gene intergroup variations

**Gene name**	**ASC (SD)**	**BMSC (SD)**
*GAPDH*	±0.3855*	±0.0780*
*GUSB*	±0.0048*	±0.0832*
*PPIA*	±0.0977*	±0.1028*
*RPL13*	±0.2843*	±0.2865*
*TBP*	±0.0090*	±0.0016*
*YWHAZ*	±0.1832*	±0.0435*

Difference in mean expression of each gene between treated and untreated groups is shown as standard deviations (SD) in unit cycles. Genes were excluded at a cutoff value above 0.2. Those with a SD below the threshold value (*) were included considered for further processing. 18S rRNA: 18S ribosomal RNA, ACTB: beta-actin, EF1-a: Elongation factor 1-alpha, GAPDH: gluco phosphate dehydrogenase, GUSB: Beta-glucuronidase, PPIA: Peptidylprolyl isomerase A, RPL13a: Ribosomal protein L13-alpha, TBP: TATA box binding protein, YWHAZ: Tyrosine 3/tryptophan 5-monooxygenase activation protein. Genes not indicated with a star (*) were deactivated in GenEx.

#### Step 3: Final output from geNorm and Normfinder

**Figure 3:**
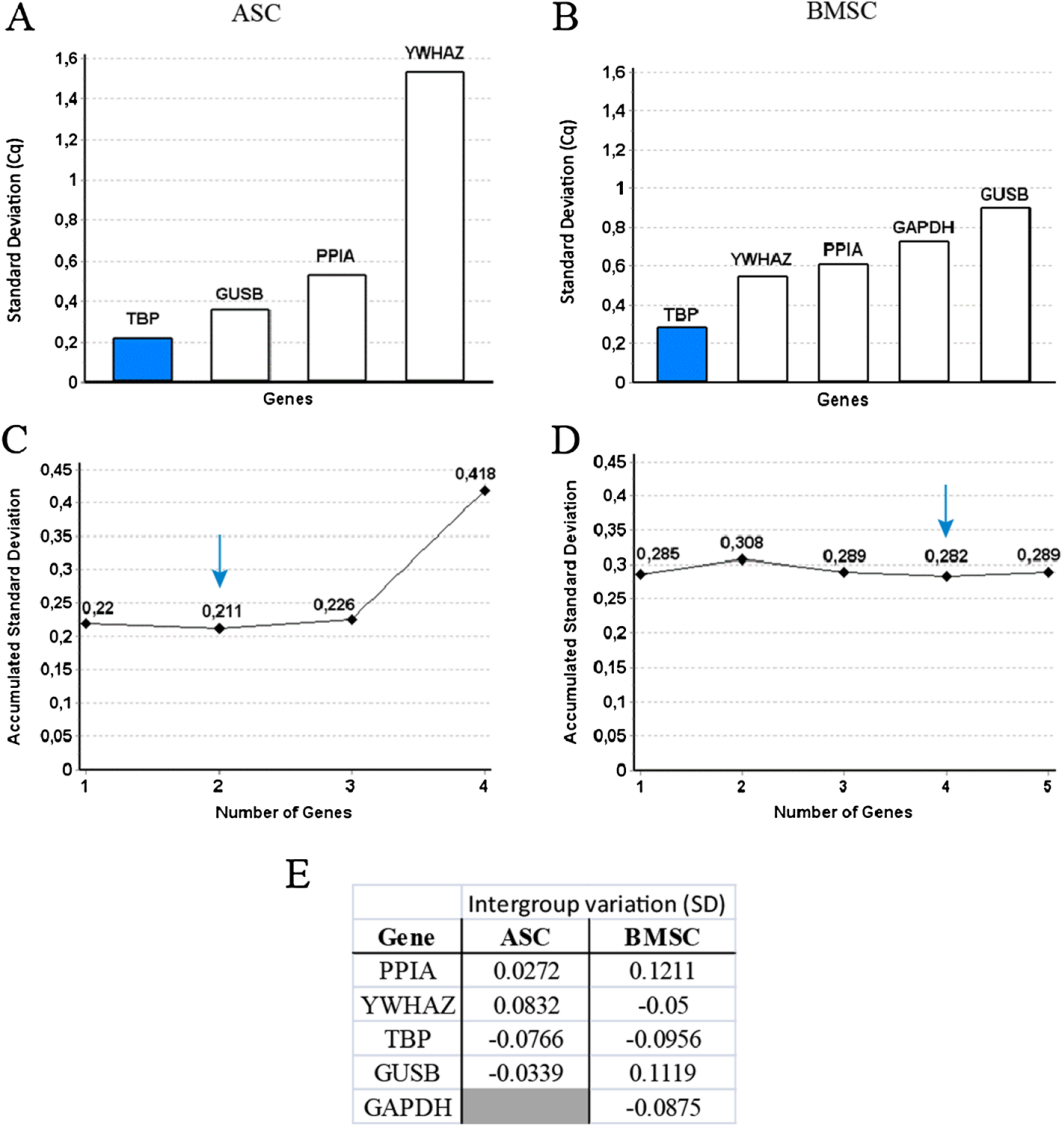
**Determination of most stable gene and optimal number of RGs.** In the third step of RG selection, the Normfinder algorithm was used on the genes remaining following the first and second eliminations. Gene ranking according to stability through VEGF treatment of ASCs **(A)** and BMSCs **(B)**. Accumulated SDs indicate the best number of RGs for ASCs **(C)** and BMSCs **(D)**. The intergroup treatment variation as calculated by Normfinder during Step 3 of the RG selection process **(E)**. The shaded histograms present the single most stable reference gene. ASCs; adipose-derived stromal cells, BMSC; bone marrow-derived stromal cells, SD; standard deviation. N = 8.

Due to the selection in step 2, the software should not regard the effect of treatment in the next step of the selection. This means that the final GenEx comparisons encompass both intragroup donor variation and intergroup treatment variation. According to Normfinder ranking order, *TBP* was found to be the most stable RG candidate through the VEGF treatment for both ASCs and BMSCs (Figure 3A and 3B). The optimal number of RGs was two for ASCs and four for BMSCs based on the accumulated SDs, which were reduced slightly by 0.009 and 0.003 respectively when compared to the use of a single RG (Figure 3C and 3D). In order to determine which RGs to use in combination we utilize intergroup treatment variation data calculated by Normfinder using only the remaining genes from Step 3 (Figure 3E). The sum of the different possible combinations were calculated and compared. The chosen combinations of *TBP* and *YWHAZ* for ASCs and *TBP, GAPDH, PPIA* and *GUSB* for BMSCs yielded a RG variation sum closest to zero.

**Figure 4:**
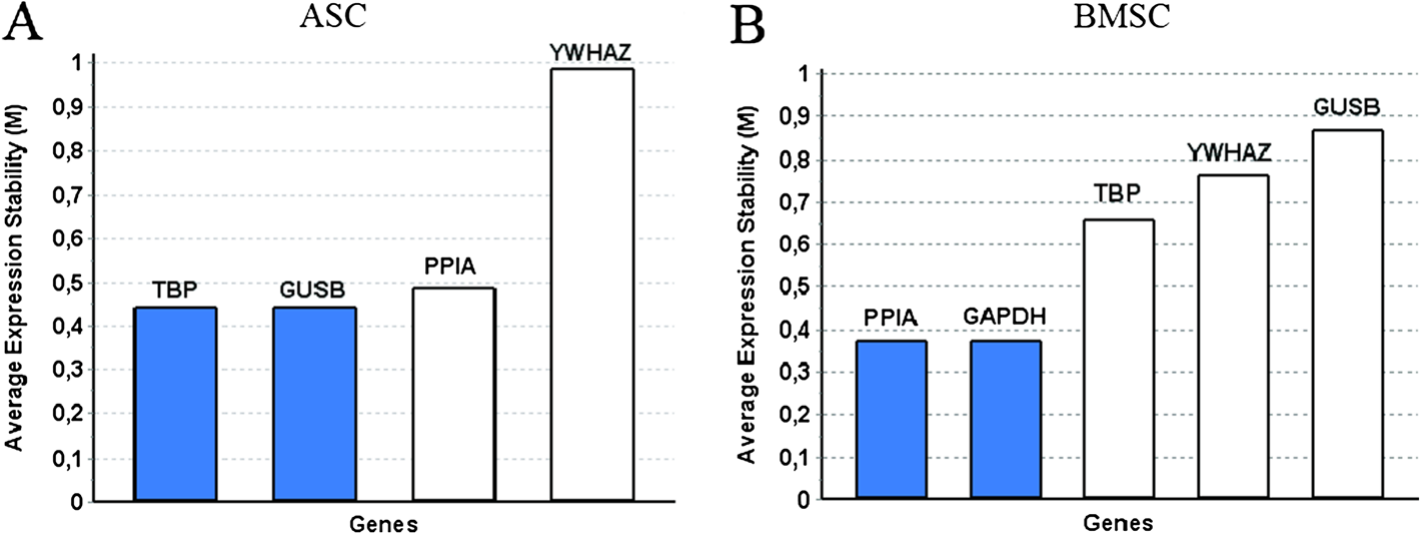
**Determination of the best correlated genes.** Average expression stability measure (M) calculated by pairwise comparison of RGs with sequential elimination of least stable RG by geNorm for ASCs **(A)** and BMSCs **(B)**. The shaded histograms indicate the two most correlated genes. ASCs; adipose-derived stromal cells, BMSC; bone marrow-derived stromal cells, SD; standard deviation. N = 8.

GeNorm uses pair-wise comparison and sequential elimination to select the pair of RGs with highest correlation, indicated by the average expression stability (M). *TBP* and *GUSB* were found to be the best correlated RGs for ASCs and *PPIA* and *GAPDH* for BMSCs (Figure 4A and 4B) with a cut-off level at 0.5.

### RG verification

**Figure 5:**
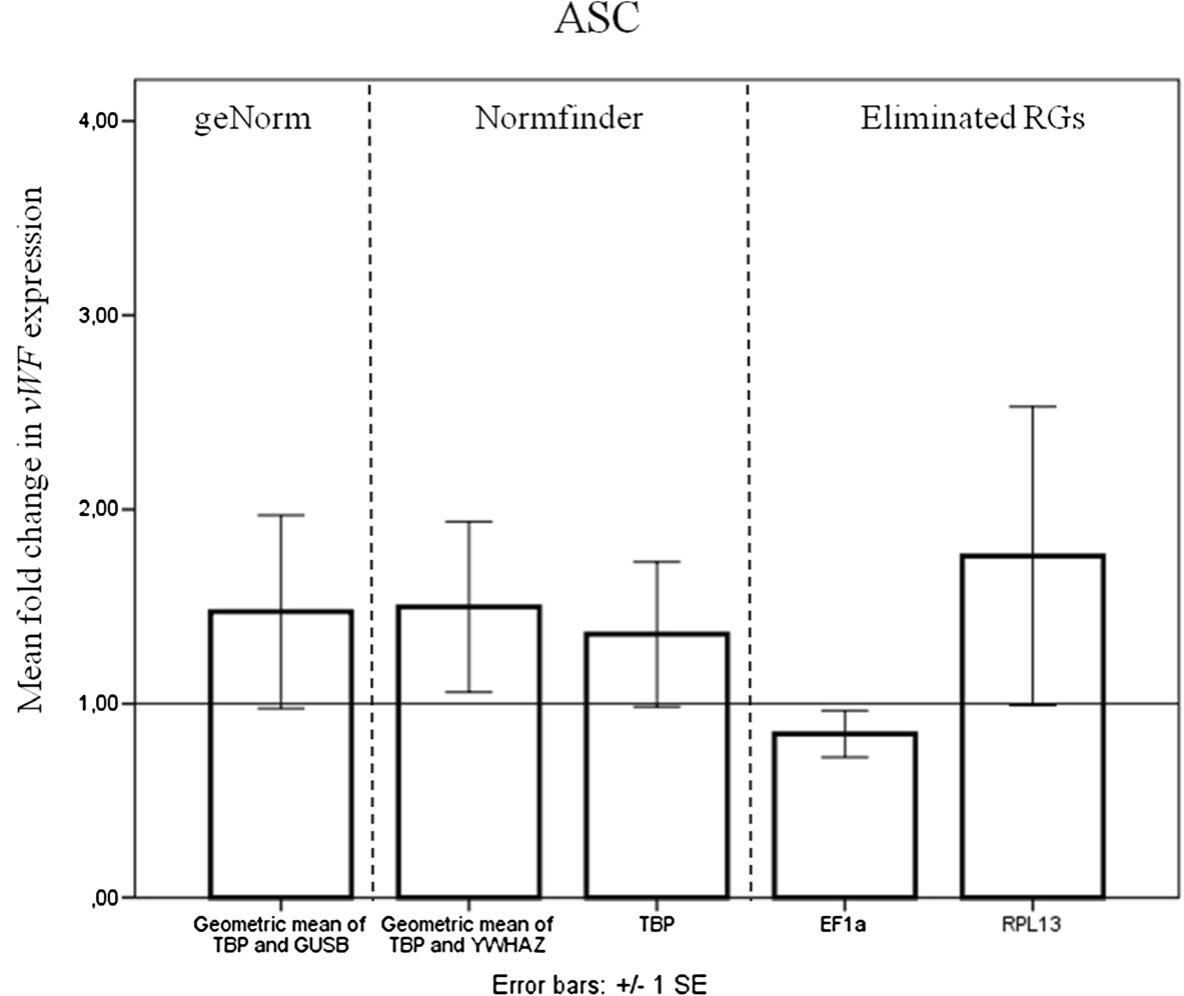
**Difference in vWF expression normalized to different RGs.** Normalization of vWF to various RGs for ASCs. The RGs were chosen from geNorm and Normfinder results. Two previously eliminated RGs, EF1-α and RPL13 are included. ASCs; adipose-derived stromal cells, RG; reference gene, vWF: von Willebrand Factor. N = 3 and error bars represent SEM.

**Figure 6:**
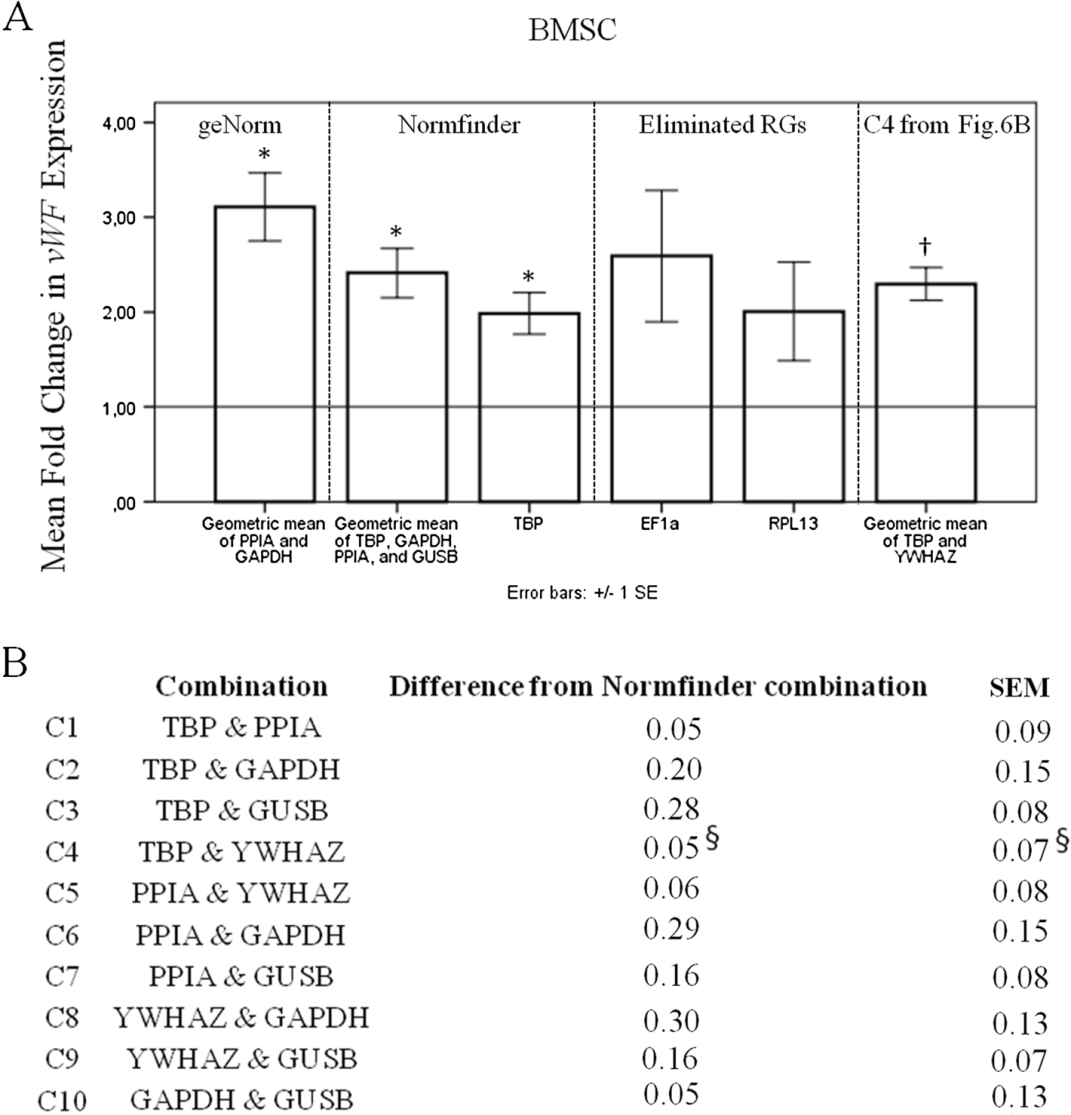
**Comparison of BMSC *vWF *expression results from two-RG-combinations.** Normalization of vWF to various RGs for BMSCs **(A)**. The RGs were chosen by the same method as those in Figure 5. *denotes significant (*p* < 0.05) *vWF* change, and † denotes significant correlation with results using the Normfinder combination of four-RGs. Test of two-RG combinations relative to results from the Normfinder combination of four genes **(B)**. 2^-∆∆Cq^ data were calculated for each RG combination using the geometric mean of the selected RGs, and compared to the mean of the results using Normfinder in the RG verification step. ^§^denotes the combination producing results most closely related to those obtained using the Normfinder four-RG combination, and the combination producing the lowest standard deviation. BMSCs; bone marrow-derived stromal cells, vWF; von Willebran Factor, RG; Reference Gene, C; Combination. N = 3 and error bars represent SEM.

We compared the effect of normalization to various RG choices on the expression of a gene of interest (GOI), *vWF,* to validate our procedure of stepwise elimination of RG candidates and to attain a final selection of the most suitable RGs for future VEGF stimulation experiments. *vWF* was chosen as positive control since it has been shown to be upregulated in other experiments by the same treatment, namely serum-deprivation combined with VEGF stimulation [19,22]. *vWF* was normalized to RGs from each selection step of our procedure. For both ASCs and BMSCs, *EF1-α* was chosen from step 1 while *RPL13* was chosen from step 2. From step 3, the single best RG found and the optimal number of RGs found by Normfinder and the two best correlated RGs given by geNorm were evaluated. Geometric mean of RG combinations were used, as suggested by *Vandesompele et al*. [14]. For ASCs, *vWF* was normalized to *TBP*, the geometric mean of *TBP* and *YWHAZ* (Normfinder), or the geometric mean of *TBP* and *GUSB* (geNorm) (Figure 5). For BMSCs, the normalization was also performed to *TBP* alone, to the geometric mean of *TBP*, *PPIA*, *GAPDH*, and *GUSB* (Normfinder), to the geometric mean of *PPIA* and *GAPDH* (geNorm) (Figure 6A). Additionally, *vWF* was normalized to the geometric mean of *TBP* and *YWHAZ* on the basis of Figure 6B.

### Poor results with normalization to eliminated RGs

For both ASCs and BMSCs, *EF1-α* was chosen from step 1 while *RPL13* was chosen from step 2 and the single best RG found by Normfinder, *TBP*, was chosen from step 3. When comparing the fold difference in *vWF* expression for ASCs, no significant increase was observed for VEGF-treated ASCs compared to controls, regardless of the RGs used for normalization. The trend in a slight upregulation of *vWF* was not investigated in depth, since this was not the aim of the study. For assessing our elimination procedure, comparison of the use of single RGs gave different results for the previously eliminated RGs compared to *TBP*. The variance in *RPL13* results was markedly larger than the variance in *TBP* results, while *EF1-α* results showed a tendency of down-regulation of *vWF*, in contrast to results from all other normalizations. For BMSC, normalizing to any of the RG combinations or *TBP* alone included in step 3 of selection, proved *vWF* to be significantly increased by the VEGF treatment. The results are not significant when normalizing to *EF1α* or *RPL13*, and their variance was greater than that for *TBP*.

### Evaluating combinations of RGs

Comparing the results from step 3 normalizations for ASCs, *TBP* alone produced results almost identical to either of the two combinations. Variance was lowest in TBP and highest in the geNorm combination, yet still almost identical (Figure 5).

For BMSCs, the fold expression increase obtained using *TBP* normalization was slightly below that for the Normfinder combination of RGs, while the geNorm combination resulted in a higher fold expression level (Figure 6A). Since the geNorm combination is the two best correlated RGs and the Normfinder combination was the best calculated number of RGs, we trust the expression level found using the Normfinder combination to be the most precise.

We further investigated whether a combination of two RGs could replace the four RG combination suggested by Normfinder for practical purposes. We found the geometric mean of *TBP* and *YWHAZ* to be the combination with the closest related and nearly identical *vWF* fold change level, also with the lowest SEM across samples (Figure 6B). This combination was compared to the others in Figure 6A. When comparing the results of *vWF* expression between the different RG combinations, only TBP is significantly different from the Normfinder combinations. However, only the results using the combination of *TBP* and *YWHAZ*, found from C4 in Figure 6B, were significantly correlated with the Normfinder combination.

## Discussion

We compared different RGs for qPCR to detect the most optimal RGs for human ASCs and BMSCs, as these cell types are currently used in clinical trials, where they are stimulated with VEGF before injection into the myocardium of patients with chronic ischemic heart disease. When investigating the effect of the growth factor on the cells on transcriptional level, it is necessary to perform RG optimization for the production of properly normalized, reliable, and reproducible results.

The best single RG for both cell types in our experimental setup was *TBP*, which has been found to be stable through several other interventions using different stem cell lines or cancer cells [15,23,24]. *TBP* and *YWHAZ* were found to be the best two-RG combination for qPCR analyses of VEGF stimulation with serum deprivation in both ASCs and BMSCs.

Only four RGs were included in the final analysis for ASCs. It is noteworthy that the reduced panel from which an RG or combination of RGs was to be selected, were the exact same RGs that *Fink et al.* have shown to have stable expression in ASCs treated with hypoxia [15]. The other candidate RGs were excluded on the basis of having intergroup variation above the 0.2 cycle cutoff or because their expression level was significantly higher in comparison to potential genes of interest. This excluded *RPL13* which was found by several groups to be the most stable RG in mesenchymal stem cells through different treatments [25,26].

Though Normfinder found the optimal number of RGs to be two (Figure 3C), and geNorm found *GUSB* to be the RG best correlated with *TBP* (Figure 4A), the difference between using *TBP* alone or in combination with *GUSB* was minimal. The inclusion of *GUSB* only leads to a reduction of 0.009 in accumulated SD (Figure 3C), and the variation in fold change of *vWF* between the two normalizations was insignificant (Figure 5). Similarly, the combination of *TBP* and *YWHAZ*, calculated from Normfinder, would give the same results as *TBP* alone. The geometric mean of this combination even had a slightly lower variance compared with that of *TBP* and *GUSB*. We suggest that *TBP* can be used alone or in combination with *YWHAZ* or *GUSB* for ASCs in this experimental setup.

For BMSCs, five RGs were included in the final analysis. The elimination explained earlier lead to the exclusion of *EF1-α*, which is considered to be a very stable RG, and the inclusion of *GAPDH*, which has been shown to be rather unstable through different treatments [15,27,28]. GeNorm identified the best combination of two genes to be *PPIA* and *GAPDH* (Figure 4B). However, the use of these two RGs leads to a higher accumulated SD (Figure 3D), and their geometric mean produced a larger fold increase in *vWF* expression compared to the other combinations (Figure 6A).

The fact that normalizing to the geometric mean of the two genes chosen by geNorm produced a larger fold increase in *vWF* compared to the other combinations is an example of geNorm calculating pair-wise correlation rather than stability when selecting the best suited RGs. The number of RGs to be used as suggested by Normfinder was the combination of four genes. The use of two RGs would increase the accumulated SD by 0.026 (Figure 3D), but due to practical reasons we further analyzed combinations of two genes to find the normalization that would produce the most similar result for *vWF* expression (Figure 6B). Since *TBP* and *YWHAZ* produced results closest to the combination of the four RGs calculated by Normfinder and displayed the lowest SEM, we obtain a combination of RGs similar to that for ASCs, which could be useful in experimental setups. Except for the use of *TBP* as single RG, none of the *vWF* expression results using the various combinations were significantly different from those using the four-RG Normfinder combination. However, the use of the other combinations could still produce Type 1 or Type 2 errors due to skewed expression levels or high degree of variance. The C4 combination from Figure 6B, *TBP* and *YWHAZ*, was the only combination significantly correlated to the four-RG Normfinder combination. This result is of course dependent on our choice of the four-RG Normfinder combination as the most stable. In contrast to BMSCs, the different RG combinations for ASCs produced more similar *vWF* expression results. This could be due to the seemingly larger impact of the VEGF treatment on BMSCs, which could likewise have larger influence on the RG expression, compared to standard culture. We have recently investigated the effect of VEGF treatment with serum-deprivation on ASCs, and found that the treatment did not produce endothelial differentiation to the same extent as shown earlier on BMSCs stimulated similarly [19,22].

The importance of using a final GOI-specific test as supplement to results from the software before choosing RGs is especially underscored by the BMSC results. Simply following the results from geNorm would have resulted in an overestimation of the VEGF effect, which could severely bias an entire study. The software is very useful in the elimination process towards the GOI-specific test, which is evident by our results with RGs eliminated in the earlier steps. The RG candidate *EF1-α* was eliminated early on in the selection process with an expression level far from that of *vWF*. For ASCs, using *EF1-α* as an RG would be misleading while in BMSCs there is a large variance. Normalizing to *RPL13* also results in a large variance for both cell types as expected, since it was eliminated early on for high intergroup variation. The fact that *EF1-α* and *RPL13* would be unsuitable to use as RGs supports our RG selection procedure.

## Conclusions

For reliable and reproducible qPCR analyses, selection of the correct RGs for a given experimental setup is of prime importance. We have presented a step-by-step procedure for selecting RGs, with emphasis on a follow-up test with a gene of interest as the final step in the selection process. Genes were eliminated based on high intergroup variations, after which Normfinder and geNorm were applied for further selection, with a final comparison of *vWF* expression levels for optimal RG selection. We found that the combination of *TBP* and *YWHAZ* as RGs is stable for normalization of VEGF-stimulated ASCs and BMSCs.

## Methods

### Experimental design

ASCs were isolated from lipoaspirate obtained from eight healthy donors of which one was male and seven were female (age between 28–57 years; mean age 43.4). BMSCs were obtained from bone marrow aspirate from eight healthy donors, two males and six females (age 20–46 years; mean age 27.8). The use of ASCs and BMSCs from healthy volunteers was approved by the National Ethical Committee protocol no. H-3-2009-119. All donors agreed to and signed the informed consent. After isolation and cultivation, ASCs and BMSCs were both stimulated with VEGF and compared with untreated control.

### Bone marrow preparation and BMSC isolation

50 ml bone marrow aspirate was obtained from the iliac crest by needle aspiration under local anesthesia. The sample was diluted 1:2 with phosphate-buffered saline pH 7.4 (PBS, −Ca^2+^ -Mg^2+^, Gibco, Invitrogen, Denmark, cat.no. 10010–015). Mononuclear cells (MNCs) were harvested by gradient centrifugation on Lymphoprep (1077 g/cm^3^, Medinor, Denmark, cat.no. 1114547), washed with PBS and counted using NucleoCounter® NC-100™ (Chemometec, Denmark) according to manufacturer’s instructions. Primary cell cultures of MNCs were established by seeding 2 × 10^7^ cells/T75-flask (Nunc, Thermo Scientific, Denmark, cat.no. 156494) in complete medium containing Dulbecco’s Modified Eagle Medium, low glucose (1 g/l) (DMEM) supplemented with 25 mM HEPES and L-Glutamin, (PAA Laboratories, Austria, cat.no. E15-808), 10% Fetal Bovine Serum Farma grade (FBS, PAA Laboratories, cat.no. A11-512) and 1% Penicillin/Streptomycin (Gibco, cat.no. 15140–122). The cells were incubated in standard conditions at 37°C in humid air with 5% CO_2_. The medium was changed 5 days after initial seeding, and subsequently every 3–4 days.

### Lipoaspirate preparation and ASC isolation

Approximately 100 ml lipoaspirate was obtained from liposuctions of subcutaneous abdominal fat performed under local anesthesia. The lipoaspirate was washed twice with PBS to remove residual blood. The adipose tissue was digested by incubation with collagenase (Collagenase NB4 (0.6 PZ U/ml, Serva GmbH, Germany) dissolved in HBSS (+CaCl_2_ + MgCl_2,_ GIBCO, cat.no. 14065–049) diluted to a concentration of 2 mM Ca^2+^) at 37°C for 45 min. under constant rotation. The collagenase was neutralized with complete medium and the suspension was filtered through a 100 μl mesh (Cell Strainer, BD Falcon, cat.no. 352360); centrifuged at 1200 g for 10 min. at room temperature, re-suspended and counted. MNCs were seeded at a density of 4.5x10^6^ cells/T75-flask in complete medium and incubated at standard conditions. After two days in culture, cells were washed with PBS to remove non‒adhering leukocytes, and complete medium added anew with subsequent change of media every three-four days.

### Cell culture

When the culture reached a confluence level of approximately 90%, cells were washed with PBS, detached with 3 ml TrypLE® (TrypLE® Select, Gibco, cat.no. 12563–029) for 10 min. at 37°C and neutralized with 7 ml complete medium. The suspension was centrifuged at 300 g for 5 min. at room temperature, counted, and seeded at 3.5x10^5^ cells/T75-flask for the experimental setup. The cells exhibited stem cell characteristics, by adhering to plastic, expressing stem cell markers and being able to differentiate [22,29-31].

### Stimulation with VEGF

BMSCs and ASCs were cultured in complete medium until their confluence was estimated to be 80%. They were then either kept in complete medium (control), or changed to serum‒deprived medium (DMEM + 2%FBS + 1%P/S) added 50 ng/ml recombinant human VEGF‒A_165_ (rhVEGFA_165_, R&D Systems, USA, cat.no. 293-VE-CF). All media was renewed every two-three days and cells were cultured for one week after which they were harvested for further processing.

### Nucleic acid extraction

Total RNA was extracted using Qiagen RNeasy® Mini Kit (Qiagen Hamburg GmbH, Hamburg, Germany, cat.no. 74106) according to the manufactures protocol. 1 ml syringe (Omnific‒F 1 ml, B.Braun Melsunger AG, Germany, cat.no. 300013) was used to homogenize the lysed cells before applying the Qiagen protocol. RNA purity was measured using NanoDrop® 1000 Spectrophotometer (Thermo Scientific, MA, USA), and the eluate was stored at −80°C. RNA purity and integrity were confirmed using RNA Nano Chips (Agilent, Santa Clara, CA, cat.no. 5067–1521) and the Agilent 2100 Bioanalyzer by following instructions of the Agilent RNA 6000 Nano Kit.

### Reverse transcription

The cDNA synthesis reaction was prepared using AffinityScript (Stratagene, Denmark, cat.no. 600559) in an eight‒tube strip (0.1 mL, MicroAmp™, Appied Biosystems®, Invitrogen, cat.no. 4358293) on ice. The total reaction volume was 20 μl with 0.5 μg RNA, 10 μl cDNA synthesis master mix, 3 μl Oligo (dT) primer, 1 μl AffinityScript RT RNase block enzyme mixture, and RNAse-DNAse free water to 20 μl total volume. Tubes were closed with caps (Applied Biosystems, cat.no. N801-0535) and the reactions were performed with an initial stage of 25°C for 5 min., 42°C for 45 min. and 95°C for 5 min. (Veriti 96 well fast thermal cycler, Applied Biosystems model no. 9901). Following synthesis, the cDNA was stored in aliquots at −20°C.

### Quantiative real-time PCR

qPCR was performed in triplicate per donor for each group within the same qPCR-run. A calibration curve was run simultaneously on the RG candidate tested on the donors. Only data obtained from runs fulfilling the same criteria for efficiency and correlation coefficient as the primer verification was included for analysis. The Cq threshold was set to the value of 0.1 for all qPCR runs. Brilliant II SYBR®green QPCR Low ROX master mix (Agilent, cat.no. 600806) was used with a total reaction volume of 25 μl in 96-well optical reaction plates (Agilent, cat.no. 401333) with 5 μl of diluted cDNA. The plate was sealed with optical plastic caps (Agilent, cat.no. 401425). qPCR was performed using Mx3000 (Stratagene) and the results were collected using Mx3000 version 4.0 software for Windows (Stratagene). The reaction was initiated by heating to 95°C for 10 min., followed by 40 cycles elongation at 60°C for 1 min. and denaturation at 95°C for 30 sec.

To verify the chosen RGs, a normalization experiment was set up. *vWF* was used as target gene, and normalized to different combinations of RG candidates. The level and the standard deviations of the fold changes between VEGF treated cells and controls were compared for normalization to different RG combinations. The fold changes in *vWF* expression between VEGF treatment and controls were calculated with the 2^-∆∆Cq^-method.

### Gel electrophoresis

A 3% 3–1 NuSieve agarose gel (Lonza, Switzerland, cat.no. 50090) was made according to the laboratory protocol with 1x TAE buffer diluted from 50x TAE buffer (Qiagen, cat.no. 129237). For visualization of qPCR products, 10% non-toxic GelStar Nucleic Acid Gel Stain (Lonza, cat.no. 50535) was added to the melted agarose before pouring the solution into a plastic well with combs. After gel solidification the combs were removed and the solid gel transferred to an electrophoresis tank (BioRad, CA, USA) and covered with 1 x TAE buffer. For every 8 μl PCR product, 2 μl Gelpilot 5x loading dye (Qiagen, cat.no. 1037650) was added and the 10 μl sample is loaded by directly injecting into the wells. A negative control was included to show no contamination of the product. Gelpilot 1 kb ladder (Qiagen, cat.no. SM0318) were used. The gel was run between 70 and 100 volt in 45 min.– 60 min. and visualized under ultraviolet light.

### Data analysis

Step 1. Cq values were plotted in Microsoft Excel and assessed visually for outliers in the lower end.

For further analysis, we used the GenEx software (MultiD Analyses AB, Sweden), and more specifically its algorithms Normfinder and geNorm. In short, Normfinder selects the most suitable RG based on the standard deviations (SDs) for intragroup and intergroup variation, while geNorm compares the RGs pair-wise and performs sequential elimination resulting in the best correlated RGs. Average Cq-values of the technical triplicates were used for analysis.

Step 2. For each cell type, the mean Cq-values of all RG candidates from all donors were entered in GenEx for the treatment and control groups.

Step 3. The function Normfinder was performed taking group classification of treatment into account, in order to verify that the candidate RGs were stably expressed and not regulated by treatment. The RG candidates with SDs reaching or exceeding 0.2 were removed from the panel by inactivation in the Data Manager of GenEx before proceeding to the next step. Normfinder was run again, without taking groups into account to give the best estimate of the genes’ stabilities and an assessment of the optimal number of RGs. Lastly, geNorm was run to determine the best correlated RGs. The ∆∆Cq-method was used when assessing the effect of VEGF treatment on the expression of *vWF* normalized to various RGs, with subsequent use of paired t-test and Pearson’s Correlation in IBM SPSS. Results were considered significant at *p*-values below 0.05.

## Abbreviations

ASC: Adipose-derived stromal cell; BMSC: Bone-marrow derived stromal cell; RG: Reference gene; VEGF: Vascular endothelial growth factor; MIQE: Minimum Information for Publication of Quantitative Real-Time PCR Experiments; RIN: RNA integrity number; 18S rRNA: 18S ribosomal RNA; ACTB: Beta-actin; EF1-α: Elongation factor 1-alpha; GAPDH: Gluco phosphate dehydrogenase; GUSB: Beta-glucuronidase; PPIA: Peptidylprolyl isomerase A; RPL13a: Ribosomal protein L13-alpha; TBP: TATA box binding protein; YWHAZ: Tyrosine 3/tryptophan 5-monooxygenase activation protein; vWF: von Willebrand factor; SD: Standard deviation; GOI: gene of interest.

## Competing interests

The authors declare that they have no competing interests.

## Authors’ contributions

JT and BFL carried out the experiments, participated in the design of the study, performed the statistical analysis and drafted the manuscript. AE participated in the design of the study and was involved with revising the manuscript critically. JK initiated the study and revised the manuscript critically. MHS conceived of the study and contributed in the design and coordination of the study. All authors read and approved the final manuscript.

## Supplementary Material

Additional file 1: Table S1 RNA Quality. **Table S2.** Primer Quality. **Figure S1.** Dissociation Curves. Click here for file

## References

[B1] Abdel-Latif A Bolli R Tleyjeh IM Montori VM Perin EC Hornung CA Zuba-Surma EK Al-Mallah M Dawn B Adult bone marrow-derived cells for cardiac repair: a systematic review and meta-analysis Arch Intern Med 2007 167 989 997 17533201 10.1001/archinte.167.10.989

[B2] Qayyum AA Haack-Sorensen M Mathiasen AB Jorgensen E Ekblond A Kastrup J Adipose-derived mesenchymal stromal cells for chronic myocardial ischemia (MyStromalCell Trial): study design Regen Med 2012 7 421 428 22594332 10.2217/rme.12.17

[B3] Zhu Y Liu T Song K Fan X Ma X Cui Z Adipose-derived stem cell: a better stem cell than BMSC Cell Biochem Funct 2008 26 664 675 18636461 10.1002/cbf.1488

[B4] Puissant B Barreau C Bourin P Clavel C Corre J Bousquet C Taureau C Cousin B Abbal M Laharrague P Penicaud L Casteilla L Blancher A Immunomodulatory effect of human adipose tissue-derived adult stem cells: comparison with bone marrow mesenchymal stem cells Br J Haematol 2005 129 118 129 15801964 10.1111/j.1365-2141.2005.05409.x

[B5] Gimble JM Katz AJ Bunnell BA Adipose-derived stem cells for regenerative medicine Circ Res 2007 100 1249 1260 17495232 10.1161/01.RES.0000265074.83288.09 PMC5679280

[B6] Ghannam S Bouffi C Djouad F Jorgensen C Noel D Immunosuppression by mesenchymal stem cells: mechanisms and clinical applications Stem Cell Res Ther 2010 1 2 20504283 10.1186/scrt2 PMC2873698

[B7] Gnecchi M Zhang Z Ni A Dzau VJ Paracrine mechanisms in adult stem cell signaling and therapy Circ Res 2008 103 1204 1219 19028920 10.1161/CIRCRESAHA.108.176826 PMC2667788

[B8] Uemura R Xu M Ahmad N Ashraf M Bone marrow stem cells prevent left ventricular remodeling of ischemic heart through paracrine signaling Circ Res 2006 98 1414 1421 16690882 10.1161/01.RES.0000225952.61196.39

[B9] Caplan AI Adult mesenchymal stem cells for tissue engineering versus regenerative medicine J Cell Physiol 2007 213 341 347 17620285 10.1002/jcp.21200

[B10] Bustin SA Benes V Garson JA Hellemans J Huggett J Kubista M Mueller R Nolan T Pfaffl MW Shipley GL Vandesompele J Wittwer CT The MIQE guidelines: minimum information for publication of quantitative real-time PCR experiments Clin Chem 2009 55 611 622 19246619 10.1373/clinchem.2008.112797

[B11] Chervoneva I Li Y Schulz S Croker S Wilson C Waldman SA Hyslop T Selection of optimal reference genes for normalization in quantitative RT-PCR BMC Bioinformatics 2010 11 253 20470420 10.1186/1471-2105-11-253 PMC2889935

[B12] Stern-Straeter J Bonaterra GA Hormann K Kinscherf R Goessler UR Identification of valid reference genes during the differentiation of human myoblasts BMC Mol Biol 2009 10 66 19573231 10.1186/1471-2199-10-66 PMC2714309

[B13] Dheda K Huggett JF Chang JS Kim LU Bustin SA Johnson MA Rook GA Zumla A The implications of using an inappropriate reference gene for real-time reverse transcription PCR data normalization Anal Biochem 2005 344 141 143 16054107 10.1016/j.ab.2005.05.022

[B14] Vandesompele J De Preter K Pattyn F Poppe B Van Roy N De Paepe A Speleman F Accurate normalization of real-time quantitative RT-PCR data by geometric averaging of multiple internal control genes Genome Biol 2002 3 7 1 0034.11 RESEARCH0034 10.1186/gb-2002-3-7-research0034 PMC126239 12184808

[B15] Fink T Lund P Pilgaard L Rasmussen JG Duroux M Zachar V Instability of standard PCR reference genes in adipose-derived stem cells during propagation, differentiation and hypoxic exposure BMC Mol Biol 2008 9 98 18976469 10.1186/1471-2199-9-98 PMC2585587

[B16] Yoon CH Koyanagi M Iekushi K Seeger F Urbich C Zeiher AM Dimmeler S Mechanism of improved cardiac function after bone marrow mononuclear cell therapy: role of cardiovascular lineage commitment Circulation 2010 121 2001 2011 20421519 10.1161/CIRCULATIONAHA.109.909291

[B17] Friis T Haack-Sorensen M Mathiasen AB Ripa RS Kristoffersen US Jorgensen E Hansen L Bindslev L Kjaer A Hesse B Dickmeiss E Kastrup K Mesenchymal stromal cell derived endothelial progenitor treatment in patients with refractory angina Scand Cardiovasc J 2011 45 161 168 21486102 10.3109/14017431.2011.569571

[B18] Haack-Sorensen M Friis T Mathiasen AB Jorgensen E Hansen L Dickmeiss E Ekblond A Kastrup J Direct intramyocardial mesenchymal stromal cell injections in patients with severe refractory angina - one year follow-up Cell Transplant 2013 22 3 521 528 22472086 10.3727/096368912X636830

[B19] Haack-Sorensen M Friis T Bindslev L Mortensen S Johnsen HE Kastrup J Comparison of different culture conditions for human mesenchymal stromal cells for clinical stem cell therapy Scand J Clin Lab Invest 2008 68 192 203 17852829 10.1080/00365510701601681

[B20] Olsson AK Dimberg A Kreuger J Claesson-Welsh L VEGF receptor signalling - in control of vascular function Nat Rev Mol Cell Biol 2006 7 359 371 16633338 10.1038/nrm1911

[B21] Andersen CL Jensen JL Orntoft TF Normalization of real-time quantitative reverse transcription-PCR data: a model-based variance estimation approach to identify genes suited for normalization, applied to bladder and colon cancer data sets Cancer Res 2004 64 5245 5250 15289330 10.1158/0008-5472.CAN-04-0496

[B22] Follin B Tratwal J Haack-Sorensen M Elberg JJ Kastrup J Ekblond A Identical effects of VEGF and serum-deprivation on phenotype and function of adipose-derived stromal cells from healthy donors and patients with ischemic heart disease J Transl Med 2013 11 219 24047149 10.1186/1479-5876-11-219 PMC3852830

[B23] Veazey KJ Golding MC Selection of stable reference genes for quantitative rt-PCR comparisons of mouse embryonic and extra-embryonic stem cells PLoS One 2011 6 e27592 22102912 10.1371/journal.pone.0027592 PMC3213153

[B24] Valente V Teixeira SA Neder L Okamoto OK Oba-Shinjo SM Marie SK Scrideli CA Paco-Larson ML Carlotti CG Jr Selection of suitable housekeeping genes for expression analysis in glioblastoma using quantitative RT-PCR BMC Mol Biol 2009 10 17 19257903 10.1186/1471-2199-10-17 PMC2661085

[B25] Curtis KM Gomez LA Rios C Garbayo E Raval AP Perez-Pinzon MA Schiller PC EF1alpha and RPL13a represent normalization genes suitable for RT-qPCR analysis of bone marrow derived mesenchymal stem cells BMC Mol Biol 2010 11 61 20716364 10.1186/1471-2199-11-61 PMC2931506

[B26] Wang Y Han Z Yan S Mao A Wang B Ren H Chi Y Evaluation of suitable reference gene for real-time PCR in human umbilical cord mesenchymal stem cells with long-term in vitro expansion In Vitro Cell Dev Biol Anim 2010 46 595 599 20440577 10.1007/s11626-010-9318-y

[B27] Everaert BR Boulet GA Timmermans JP Vrints CJ Importance of suitable reference gene selection for quantitative real-time PCR: special reference to mouse myocardial infarction studies PLoS One 2011 6 e23793 21858224 10.1371/journal.pone.0023793 PMC3157472

[B28] Condori J Nopo-Olazabal C Medrano G Medina-Bolivar F Selection of reference genes for qPCR in hairy root cultures of peanut BMC Res Notes 2011 4 392 21985172 10.1186/1756-0500-4-392 PMC3199266

[B29] Friis T Haack-Soorensen M Hansen SK Hansen L Bindslev L Kastrup J Comparison of mesenchymal stromal cells from young healthy donors and patients with severe chronic coronary artery disease Scand J Clin Lab Invest 2011 71 193 202 21222501 10.3109/00365513.2010.550310

[B30] Bourin P Bunnell BA Casteilla L Dominici M Katz AJ March KL Redl H Rubin JP Yoshimura K Gimble JM Stromal cells from the adipose tissue-derived stromal vascular fraction and culture expanded adipose tissue-derived stromal/stem cells: a joint statement of the International Federation for Adipose Therapeutics and Science (IFATS) and the International Society for Cellular Therapy (ISCT) Cytotherapy 2013 15 641 648 23570660 10.1016/j.jcyt.2013.02.006 PMC3979435

[B31] Dominici M Le Blanc K Mueller I Slaper-Cortenbach I Marini F Krause D Deans R Keating A Prockop D Horwitz E Minimal criteria for defining multipotent mesenchymal stromal cells. The International Society for Cellular Therapy position statement Cytotherapy 2006 8 315 317 16923606 10.1080/14653240600855905

