# Supplementary material for: Identification of a common reference gene pair for qPCR in human mesenchymal stromal cells from different tissue sources treated with VEGF
Source: BMC Mol Biol. 2014 May 28;15:11. doi: 10.1186/1471-2199-15-11 (PMC4045907; doi:10.1186/1471-2199-15-11)
Supplement: Additional file 1: Table S1 — RNA Quality. Table S2. Primer Quality. Figure S1. Dissociation Curves. [file 1471-2199-15-11-S1.docx]

**Table 1S – RNA Quality**

| **Donor** | **RIN (c)** | **RIN (s)** | **[RNA] (ng/µl) (c)** | **[RNA] (ng/µl) (s)** | **A260/280 (c)** | **A260/280 (s)** |
| --- | --- | --- | --- | --- | --- | --- |
| ASC 1 | 10 | 10 | 433 | 324 | 2.0 | 2.0 |
| ASC 2 | 10 | 10 | 212 | 66 | 2.1 | 2.0 |
| ASC 3 | 10 | 10 | 65 | 219 | 2.0 | 2.0 |
| ASC 4 | 10 | 10 | 677 | 185 | 2.1 | 2.1 |
| ASC 5 | 10 | 10 | 859 | 467 | 2.1 | 2.0 |
| ASC 6 | 10 | 10 | 718 | 315 | 2.0 | 2.0 |
| ASC 7 | 10 | 9.8 | 399 | 140 | 2.0 | 2.1 |
| ASC 8 | 10 | 10 | 1143 | 575 | 2.1 | 2.1 |
| BMSC 1 | 10 | 10 | 904 | 783 | 2.1 | 2.1 |
| BMSC 2 | 10 | 10 | 1213 | 777 | 2.1 | 2.1 |
| BMSC 3 | 10 | 10 | 623 | 425 | 2.1 | 2.0 |
| BMSC 4 | 10 | 10 | 612 | 837 | 2.1 | 2.1 |
| BMSC 5 | 10 | 10 | 741 | 782 | 2.1 | 2.1 |
| BMSC 6 | 10 | 10 | 954 | 876 | 2.1 | 2.1 |
| BMSC 7 | 10 | 10 | 1420 | 1018 | 2.1 | 2.1 |
| BMSC 8 | 10 | 10 | 725 | 1074 | 2.1 | 2.1 |

Values for several measures of RNA quality and integrity, including RIN, RNA concentration, and absorbance ratios A260/280 for protein contamination. Values are indicated for donor ASCs and BMSCs that were cultured in complete medium (c) or stimulated with VEGF (s). ASCs; adipose-derived stromal cells, BMSCs; bone marrow-derived stromal cells, RIN; RNA integrity number.

**Table 2S – Primer Quality**

| **Primer** | **Efficiency** | **Correlation coefficient** |
| --- | --- | --- |
| *18SrRNA* | 102 % | 0.997 |
| *ACTB* | 108 % | 0.994 |
| *EF1a* | 108 % | 0.994 |
| *GAPDH* | 98 % | 0.994 |
| *GUSB* | 105 % | 0.997 |
| *PPIA* | 102 % | 0.998 |
| *RPL13a* | 102 % | 0.999 |
| *TBP* | 98 % | 0.986 |
| *YWHAZ* | 101 % | 0.990 |
| *vWF* | 95 % | 0.992 |

Efficiencies and corresponding correlation coefficients, R^2^, of primers for the panel of reference genes and gene of interest. 18S rRNA: 18S ribosomal RNA, ACTB: beta-actin, EF1-a: Elongation factor 1-alpha, GAPDH: gluco phosphate dehydrogenase, GUSB: Beta-glucuronidase, PPIA: Peptidylprolyl isomerase A, RPL13a: Ribosomal protein L13-alpha, TBP: TATA box binding protein, YWHAZ: Tyrosine 3/tryptophan 5-monooxygenase activation protein, vWF: von Willebrand Factor.

**Figure 1S - Dissociation Curves**


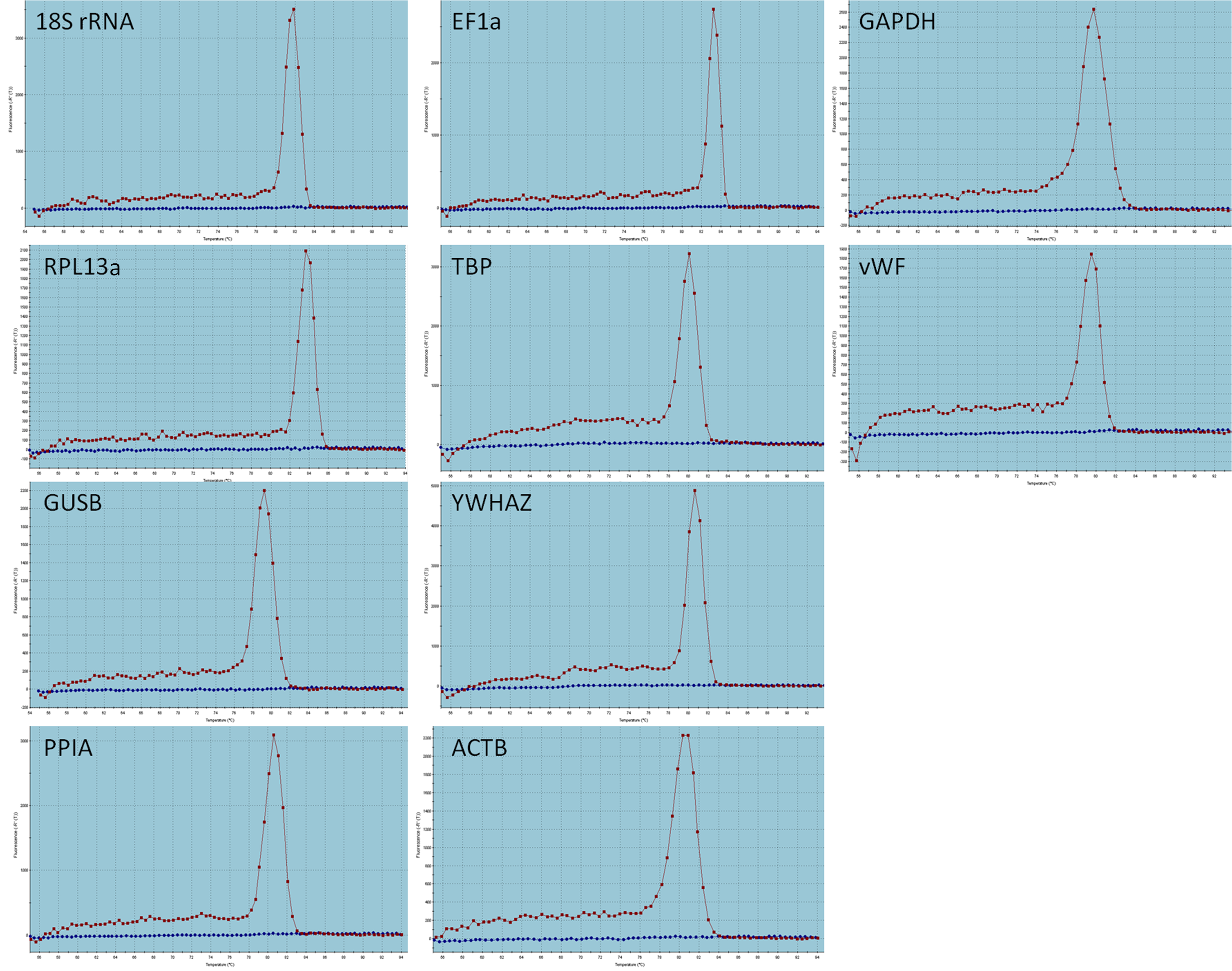


Representative dissociation curves for each primer set. The x-axis shows degree increases in temperature from 56 to 94 degrees Celsius. Change in fluorescence/change in temperature is plotted against temperature. 18S rRNA: 18S ribosomal RNA, ACTB: beta-actin, EF1-a: Elongation factor 1-alpha, GAPDH: gluco phosphate dehydrogenase, GUSB: Beta-glucuronidase, PPIA: Peptidylprolyl isomerase A, RPL13a: Ribosomal protein L13-alpha, TBP: TATA box binding protein, YWHAZ: Tyrosine 3/tryptophan 5-monooxygenase activation protein, vWF: von Willebrand Factor. Red curve = SYBR Green signal, blue = ROX reference dye.
